# Differences in Demographic, Risk, and Protective Factors in a Clinical Sample of Children who Experienced Sexual Abuse Only vs. Poly-victimization

**DOI:** 10.3389/fpsyt.2021.789329

**Published:** 2022-01-12

**Authors:** Nicole Racine, Jenney Zhu, Cailey Hartwick, Sheri Madigan

**Affiliations:** ^1^Department of Psychology, University of Calgary, Calgary, AB, Canada; ^2^Alberta Children's Hospital Research Institute, Calgary, AB, Canada; ^3^Child Abuse Service, Alberta Children's Hospital, Alberta Health Services, Calgary, AB, Canada

**Keywords:** sexual abuse, protective factor, risk factor, poly-victimization, child

## Abstract

Children exposed to child sexual abuse (CSA) vary considerably with regards to their presenting concerns and treatment needs. One factor creating heterogeneity amongst children experiencing CSA is their history of experiencing other victimizations (i.e., poly-victimized or not). However, little is known about risk factors for poly-victimization as well as differences in protective factors among these two groups. Additionally, there is currently limited understanding of whether poly-victimization is associated with greater trauma symptoms in children exposed to CSA and being seen for trauma treatment. Using a clinical sample of 117 children who were sexually abused (64 CSA only and 53 poly-victimized) ranging from age 3–18 years, the current study examined demographic characteristics, abuse characteristics, trauma symptoms, and protective factors using casefile review methodology. After accounting for other risk factors, parental abuse history and protective factors were significantly associated with child poly-victimization status. Children exposed to poly-victimization were more likely to have financial concerns $\chi_{\left(1,115\right)}^{2}$ = 4.16, *p* = 0.04, parents with abuse histories $\chi_{\left(1,117\right)}^{2}$ = 8.93, *p* = 0.003, and parents with histories of mental health or substance use difficulties $\chi_{\left(1,117\right)}^{2}$ = 4.02, *p* = 0.045. Although cumulative trauma symptoms scores were higher for children who were poly-victimized compared to CSA only, *t*_(115)_ = −2.24, *p* = 0.03, multiple regression analyses showed that poly-victimization status was not significantly associated with child trauma symptoms after accounting for other demographic and abuse characteristics. Assessing and understanding the extent to which children exposed to CSA have experienced other forms of maltreatment is critical for identifying children who may be most at risk of poor outcomes.

## Introduction

Child sexual abuse (CSA) is one of the most pernicious forms of childhood adversity. With 13% of children worldwide experiencing sexual abuse (1), the cumulative mental health and physical health consequences associated with CSA are substantial (2–5). Upwards of 50% of children develop trauma symptoms following exposure to CSA and require psychological treatment or intervention (6). Yet, the experience and subsequent treatment needs of children who have been sexually abused are varied. Several variables contribute to the presenting symptoms and treatment needs of children who have been sexually abused, including abuse severity (7), abuse frequency (8), and perpetrator characteristics (9, 10). An additional factor that may differentiate children with a history of CSA is whether or not they have also been exposed to other forms of abuse in addition to CSA, a term known as poly-victimization (11). Previous research has shown that children who have been poly-victimized are more likely to have emotional difficulties in adulthood than those exposed to CSA only and unexposed children (12). However, there is limited understanding of what risk factors may contribute to poly-victimization as well as how children exposed to poly-victimization may differ from children exposed to CSA only. Understanding factors that contribute to the differential profiles of CSA and their related experiences could inform treatment planning and decision making for children exposed to single vs. multiple forms of maltreatment.

Exposure to childhood sexual abuse is a complex and multi-faceted experience. The experience of one child who has been sexually abused can differ widely from the experience of another. Indeed, the term CSA is used to describe a wide range of experiences that vary in characteristics, such as the severity and chronicity of the abuse, relationship to the perpetrator, and age at which it occurred (13). These characteristics have been shown to be inter-related (13), with younger age of abuse onset and being abused by a family member being closely linked. These variables are also important determinants of the outcomes for children who have been sexually abused, including greater development of trauma symptoms and mental health difficulties amongst those with more severe and chronic sexual abuse, and those whose sexual abuse perpetrator was intrafamilial (8). Outcomes for children who have experienced CSA may also vary based on whether the child has been exposed to other forms of abuse. Finkelhor et al. coined the term poly-victimization, which refers to the exposure of multiple forms of victimization, including child abuse (14). Children who experience one form of abuse or maltreatment are highly likely to experience other forms of victimization (8, 15, 16) indicating that experiences of abuse co-occur and can be cumulative. This may in part be attributable to the pathways that lead to exposure to abuse and poly-victimization, such as living in a violent or chaotic family and/or neighborhood (17). In line with developmental theory on cumulative risk (18, 19), the accumulation of abuse experiences is an important consideration with regards to children's clinical presentations and may be an important determinant of children's outcomes in trauma treatment (20).

While poly-victimization has been a burgeoning area of research, limited research has examined differences in demographics, abuse characteristics, and trauma symptoms among children exposed to CSA only as compared to children exposed to CSA in addition to other forms of maltreatment. Additionally, there is also a limited understanding of the risk factors for poly-victimization in clinical samples. One study showed that children exposed to both sexual and physical abuse have more psychiatric diagnoses and mental health difficulties than children exposed to CSA only and physical abuse only (21). Another study conducted in Korea examined differences in abuse characteristics and mental health symptoms among children exposed to CSA only vs. children exposed to CSA plus other interpersonal traumas (e.g., witnessing violent crime, witnessing domestic violence) (22). They found that poly-victimized children were more likely to be older, be in single-parent families, have longer durations of CSA, and have perpetrators within the family compared to children exposed to CSA only (22). The rate of psychiatric diagnoses and severe behavior problems were also higher among children who were exposed to CSA in addition to other forms of interpersonal trauma (22). The current study builds on previous research and provides a novel contribution in three main ways: (1) we identify risk factors for poly-victimization in a community-based setting, (2) we examine whether protective factors differ among children who are poly-victimized vs. children exposed to CSA only, and (3) we examine whether poly-victimization is significantly associated with child trauma symptoms above and beyond other demographic and abuse characteristics. Our use of a community-based clinical sample makes the findings from the current study highly clinically relevant to service providers working with children exposed to CSA.

In addition to understanding differences in abuse characteristics and mental health symptoms for children exposed to CSA only vs. children who are poly-victimized, it is also important to consider differences in parental characteristics and protective factors at the outset of treatment. Parent mental health and well-being have been identified as important determinants of children's adaptation following exposure to CSA (23). That is, children who have a parent or caregiver who is emotionally supportive and available are more likely to disclose CSA (24), and also more likely to have successful treatment outcomes (25). A parent's own mental health or substance use difficulties as well as previous exposure to trauma may act as barriers in being able to provide this support. Identifying differences in protective factors is also an important consideration. For example, protective factors, including individual skills and use of social supports, have been shown to moderate the association between poly-victimization and the onset of psychological problems (26). Additionally, previous research has shown that children who have fewer protective factors are at increased risk for dropping out of trauma treatment (20). Thus, understanding whether there are differences in protective factors for children who have been poly-victimized is critical for informing treatment goals and targets.

To build on previous research, the current study sought to explore differences in demographic factors, abuse characteristics, trauma symptoms, and protective factors between children referred for trauma treatment who were exposed to CSA only vs. those who have been poly-victimized. Previous work has focused on differences in abuse characteristics as well as psychiatric diagnoses and behavior problems among these two groups (22). The current study adds to this growing knowledge base by examining differences in trauma symptoms as well as protective factors. Children included in the current study are part of a community sample of children exposed to sexual abuse and thus findings are highly clinically relevant to community practice settings treating children who have been sexually abused and their families. Understanding differences in presenting trauma symptoms and protective factors can help disentangle the unique contribution of sexual abuse vs. other experiences of maltreatment and can provide valuable clinical information in terms of which children and families may require additional supports and resources to recover from their trauma.

## Materials and Methods

### Study Design

Data for the current study were extracted from the clinical files of children referred to a child abuse treatment service in Western Canada. Files reviewed were open between the dates of January 2016 and June 2017 and the case file review occurred between March 2017 and October 2018. Using guidelines for retrospective chart review (27), demographic characteristics (i.e., child age, sex, financial concerns, parent history of abuse, and parent mental health history), abuse characteristics (i.e., perpetrator, severity, and frequency), presenting trauma symptoms, and protective factors (e.g., support from caregivers, peers, and school) were extracted from clinical files by two trained coders using a standardized data extraction protocol. All information was based on information provided in a standard intake record form, clinician reports, case notes, questionnaires completed by children and families, and any medical documents included in the case files.

### Participants

The current study was part of a larger retrospective case file review examining characteristics and treatment needs of children exposed to adversity more broadly and referred for psychosocial services at the Child Abuse Service (CAS) (28, 29). The CAS is a multi-disciplinary, psychosocial treatment team that assesses the impact of sexual abuse, as well as severe and complex physical abuse and neglect, and provides therapeutic intervention to address psychological and relational sequelae of abuse. Children are referred to the CAS from the hospital, medical clinics, community physicians, child welfare, parents/guardians, or via self-referral. Referral criteria for the CAS include: (1) exposure to sexual abuse and/or severe and complex physical abuse and/or neglect, and (2) the child is experiencing emotional, behavioral, or relational distress related to their abuse exposure, and (3) the child is under the age of 18 years at the time of referral. Children referred to and treated at the CAS are among the most severe and complex child abuse cases within the region. As described in previous research (28), more than 60% of cases are referred for a primary presenting concern of sexual abuse. As such, the sample of sexually abused children is largely representative of the population who is referred to the CAS. For 82.1% of children, the primary caregiver was the child's biological parent. In 76.1% of cases, the primary caregiver was the child's mother. It is common practice at the CAS to conduct assessments and treatments with non-offending caregivers, particularly in instances of sexual abuse. Thus, the sexual abuse perpetrator was not the primary caregiver involved with the child's treatment.

To be included in the current file review, children had to be assessed and/or treated at the CAS from January 2016 to March 2017 and were no longer receiving service (i.e., their file was closed). A total of 176 files were extracted, of which 117 had experienced sexual abuse. Sixty-four children were exposed only to sexual abuse while 53 children were exposed to sexual abuse in addition to at least one other form of maltreatment, including physical abuse, emotional abuse, physical neglect, and emotional neglect, and were thus considered to be in the poly-victimized group.

### Measures

#### Demographic Information

Demographic information was gathered from an intake form completed by an intake worker at the time of referral. The intake form was completed with the referral source who was either the biological parent (72.1%), child welfare worker (10.8%), foster parent (4.5%), or another source such as a therapist, police officer, or extended family member (12.5%). Demographic information included in the current study were the child's age, sex (male or female), and financial difficulties (i.e., considered to have financial difficulties or not). Information about whether or not one of the child's parents had a history of child abuse (yes/no) as well as a history of mental health or substance use difficulties (yes/no) was also obtained from the referral source.

#### Abuse Types and Characteristics

At the time of referral, the types of abuse the child had experienced were collected. However, if the child reported additional abuse types upon meeting the therapist, these were also captured in the file review methodology. Potential abuse types included sexual abuse, physical abuse, emotional abuse, physical neglect, and emotional neglect. Based on definitions provided in previous research (30), sexual abuse was operationalized as any of the following being committed by an adult or individual at least 5 years older than the child: touched, fondled, oral sex, anal sex, or vaginal sex. Physical abuse was operationalized as being pushed, grabbed, slapped, object thrown at the child, or hit. Emotional abuse was defined as being sworn at, insulted, put down, humiliated, or threatened. Physical neglect was defined as failure to meet a child's basic physical needs such as provision of food, clean clothes, and protection from safety. Emotional neglect was operationalized as a child often or very often not feeling loved, a sense of belonging, or support from their caregivers. All abuse types were rated as being present (1) or absent (0) for each child. A total score across abuse types was calculated and participants were categorized as either having experienced child sexual abuse only (CSA only) or as having experienced poly-victimization.

Abuse characteristics were gathered as part of the intake interview (28). The child's relationship to the perpetrator was coded as either a non-family member (0) or as a family member (1). All biological parents, non-biological parents, siblings, and extended relatives were coded as being family members. Strangers and acquaintances were coded as non-family members. Abuse invasiveness was coded using a 4-point scale. A score of 1 referred to no physical contact such as indecent exposure or exposure to pornography, a code of 2 was assigned for fondling or touching, a code of 3 was assigned for oral sex, and a code of 4 was assigned for genital or anal penetration.

#### Trauma Symptoms

Trauma symptoms at the time of referral were coded as present (coded as 1) or absent (coded as 0) based on a list of 11 symptoms derived from the DSM-5 diagnostic criteria for Post-Traumatic Stress Disorder (31). These trauma symptoms were selected as they commonly present in children who have been exposed to child abuse (32). The 11 trauma symptoms systematically asked about in the intake process were: physical aggression, oppositional behavior, acting out in a sexual manner, symptoms of anxiety or fearfulness, presence of nightmares or sleep disturbance, changes in weight or appetite, preoccupation with or can't stop thinking about the abuse or adverse event, feelings of guilt or shame related to the abuse, persistently sad or withdrawn behavior, thoughts or attempts of self-harm or suicide, and substance use. The total sum of endorsed items was calculated. Examining the internal consistency of all the items, the Cronbach's alpha was 0.61, which is considered acceptable (33). The reliability for the summed trauma symptoms score was excellent (ICC = 0.90).

#### Protective Factors

Protective factors were coded using a cumulative score based on items from the Child and Youth Resilience Measure [CYRM-28; (34)]. The CYRM is typically administered as a self-report or caregiver report questionnaire (34), however, given the retrospective file review methods employed in the current study, we extracted information on the presence or absence of eight overall subscales pertaining to the individual child (i.e., personal skills, peer support, and social skills), caregiver (i.e., physical and psychological caregiving), and context (i.e., spiritual, educational, and cultural support) (35). Each subscale was scored as either present (1) or absent (0) based on information in the child's file. For the spiritual and cultural support variables, more than 80% of participants had no available data on these factors, therefore, responses on the spiritual and cultural variables were not included in the analyses. Internal consistency in the current study was acceptable with a Cronbach's alpha of 0.66. The intraclass correlation for the reliability score among coders for the total protective factors score was 0.69, which is considered adequate.

### Data Analysis

All analyses were conducted in SPSS 25. Descriptive statistics, including means and percentages were estimated for child demographic factors. Differences in demographic, abuse, symptoms, and protective factors between children exposed to sexual abuse only vs. children who were poly-victimized were examined using chi-square tests for dichotomous variables and independent sample *t*-tests for continuous variables. Correlation and point-biserial correlations were calculated across all variables. Logistic regressions were calculated to examine what demographic characteristics and protective factors were associated with child poly-victimization status. Finally, a multiple regression analysis was conducted to examine whether poly-victimization status is significantly associated with child trauma symptoms above and beyond other demographic and abuse-related factors.

A power calculation was conducted using G^*^Power software (36) to determine the required sample size across CSA and poly-victimization groups. To detect a medium effect size (Cohen's d = 0.62), with a power of 0.95 and alpha of 0.05, an overall sample size of 116 participants (63 and 53 participants in each group) was needed. Thus, a sample size of 117 participants was adequate to detect a medium effect size in the current study.

## Results

### Descriptive Characteristics

All descriptive statistics are presented in Table 1. On average children were 10.59 years of age (Range: 3.92–17.67) and 77.8% (*n* = 91) were female. The majority of the sample did not report financial concerns (84.6%). More than half of parents in the sample had a history of child abuse themselves (54.7%) and the majority of parents (65.8%) had history of either mental illness or substance use difficulties. With regards to abuse characteristics, 55.6% of perpetrators were a family member. Frequencies of individual trauma symptoms and protective factors are presented in Table 1.

**Table 1:** Descriptive characteristics of the total sample (*n* = 117).

**Demographic characteristics**			
	**Mean (Standard Deviation)**	**Range**	***N* (%)**
Child age	10.59 (3.60)	3.92–17.67	
**Child sex**			
Male			25 (21.4)
Female			91 (77.8)
Transgendered			1 (0.9)
**Victimization status**			
CSA only			64 (54.7)
CSA plus one other form of maltreatment			21 (17.9)
CSA plus two other forms of maltreatment			14 (12.0)
CSA plus three other forms of maltreatment			11 (9.4)
CSA plus four other forms of maltreatment			7 (6.0)
**Financial concerns**			
Yes			16 (13.7)
No			99 (84.6)
**Parent child abuse history**			
Yes			64 (54.7)
No			53 (45.3)
**Parent history of mental illness or substance use difficulties**			
Yes			77 (65.8)
No			40 (34.2)
**Abuse characteristics**			
Invasiveness of abuse	2.78 (0.96)	1–4	
**Relationship to perpetrator**			
Family member			65 (55.6)
Non-family member			52 (44.4)
**Trauma symptoms**			
Cumulative score	5.17 (2.44)	0-10	
Physical aggression			55 (47.0)
Oppositional behavior			52 (44.4)
Acting out in a sexual manner			37 (31.6)
Anxiety or fearfulness			93 (79.5)
Nightmares or sleep disturbance			80 (68.4)
Changes in weight or appetite			44 (37.6)
Preoccupation with abuse			56 (47.9)
Feelings of guilt or shame			66 (56.4)
Sad or withdrawn behavior			67 (57.3)
Thoughts or attempts of self-harm or suicide			42 (35.9)
Substance use			13 (11.1)
**Protective factors**			
Cumulative score	4.97 (1.32)	1-6	
Personal skills			89 (76.1)
Peer support			66 (56.4)
Social skills			77 (65.8)
Physical caregiving			97 (82.9)
Psychological caregiving			106 (90.6)
Educational support			70 (59.8)

### Pearson and Point-Biserial Correlations Among Study Variables

Correlations among study variables are presented in Table 2. Being poly-victimized was negatively associated with the protective factors score and positively associated with trauma symptoms and invasiveness of sexual abuse. Older children had higher cumulative trauma symptoms. Cumulative trauma symptoms and the protective factors score were negatively associated. Cumulative trauma symptoms were positively associated with a parental history of abuse and a parental history of mental health or addictions issues.

**Table 2:** Pearson and point-biserial correlations among study variables.

**Variables**	**1**	**2**	**3**	**4**
1.Child age	1.0	–	–	–
2.Protective factors	0.05	1.0	–	–
3.Cumulative trauma symptoms	0.24^**^	−0.36^**^	1.0	–
4.Sexual abuse severity	0.17	−0.14	0.10	1.0
5.Child sex	−0.05	−0.09	0.05	0.16
6.Financial concerns	−0.12	−0.17	0.10	0.10
7.Family member perpetrator	0.05	−0.15	−0.11	0.09
8.Parental abuse history	0.16	−0.06	0.24^**^	0.05
9.Parental mental health or addictions history	0.06	−0.22*	0.23*	0.01
10.Child was poly–victimized	0.09	−0.47^**^	0.21*	0.30^**^

*Pearson correlations were calculated for two continuous variables. Point-biserial correlations were calculated for one continuous and one dichotomous variable. Correlations among dichotomous variables (i.e., child sex, financial concerns, family member perpetrator, parental abuse history, parental mental health or addictions history, and poly-victimization status) were not calculated*.

*correlation is significant at p < 0.05.

** *correlation is significant at p < 0.01*.

### Examining Sociodemographic Risk Factors for Poly-Victimization

Using logistic regression, we examined whether child demographic factors and protective factors were associated with exposure to poly-victimization (See Table 3). In unadjusted analyses, having financial concerns, parental abuse history, and a parental history of mental health or addiction difficulties were associated with exposure to poly-victimization. A higher protective factors score was associated with decreased likelihood of being poly-victimized. When all significant variables were simultaneously entered into a logistic regression model, only the parental abuse history and cumulative protective factors score remained significantly associated with child poly-victimization status. A total of 41.6% of the variance in poly-victimization status was accounted for by the adjusted model.

**Table 3:** Unadjusted and adjusted logistic regression analyses of risk factors for poly-victimization status.

**Variables**	**B**	**SE**	***P*-value**	**Odds Ratio**	**95% CI**
**Unadjusted analyses**					
Child age	0.05	0.05	0.35	1.05	0.95, 1.16
Protective factors	−0.90	0.23	<0.001	0.41	0.26, 0.64
Child sex	−0.04	0.46	0.93	0.96	0.39, 2.34
Financial concerns	1.14	0.58	0.049	3.11	1.01, 9.64
Parental abuse history	1.15	0.39	0.003	3.17	1.47, 6.83
Parental mental health or addictions history	0.81	0.41	0.047	2.25	1.01, 4.99
**Adjusted analyses**					
Protective factors	−0.98	0.25	<0.001	0.38	0.23, 0.62
Financial concerns	1.18	0.78	0.13	3.25	0.70, 14.99
Parental abuse history	1.47	0.66	0.03	4.33	1.19, 15.79
Parental mental health or addictions history	−0.32	0.66	0.63	0.73	0.20, 2.66

*For the unadjusted analyses, each variable was entered separately into the logistic regression model. For the adjusted analyses, significant variables from the unadjusted analyses were simultaneously entered into the logistic regression model. A total of 41.6% of the variance in poly-victimization status was accounted for by the adjusted model*.

### Demographic and Abuse Characteristic Differences

Differences in demographic and abuse characteristics for children exposed to CSA only vs. children who were poly-victimized are presented in Table 4. Children exposed to CSA only vs. children who were poly-victimized did not differ in age or sex. Children who were poly-victimized were more likely to have financial concerns in their family, $\chi_{\left(1,115\right)}^{2}$ = 4.16, *p* = 0.04. Parents of children who were poly-victimized children were more likely to have a history of childhood abuse, $\chi_{\left(1,117\right)}^{2}$ = 8.93, *p* = 0.003, as well as a history of mental health or substance use difficulties, $\chi_{\left(1,117\right)}^{2}$ = 4.01, *p* = 0.045. Children who were poly-victimized were more likely to have been abused by a perpetrator who was a family member (69.8%) compared to CSA only children (43.8%) and had higher mean sexual abuse invasiveness scores, *t*_(111)_ = −3.35, *p* = 0.001.

**Table 4:** Differences in demographic and abuse characteristics among children exposed to csa only vs. those who were poly-victimized.

**Characteristic**	**Exposure group**			
	**CSA only (*n =* 64)**	**Poly–victimization (*n =* 53)**	***t*-value**	***p*-value**
Child age (years), M (SD)	10.31 (3.65)	10.93 (3.54)	−0.93	0.36
Abuse invasiveness, M (SD)	2.52 (0.86)	3.10 (0.98)	−3.35	0.001
			***χ^2^***	***P*** **–value**
**Child sex**, ***N*** **(%)**				
Male	14 (21.9)	11 (21.2)	0.009	0.93
Female	50 (78.1)	41 (78.8)		
**Financial concerns**, ***N*** **(%)**				
No	58 (92.1)	41 (78.8)	4.16	0.04
Yes	5 (7.9)	11 (21.2)		
**Parental child abuse history**, ***N*** **(%)**				
No	37 (57.8)	16 (30.2)	8.93	0.003
Yes	27 (42.2)	37 (69.8)		
**Parental history of mental illness or substance use difficulties**, ***N*** **(%)**				
No	27 (42.2)	13 (24.5)	4.02	0.045
Yes	37 (57.8)	40 (75.5)		
**Relationship to perpetrator**, ***N*** **(%)**				
Family member	28 (43.8)	37 (69.8)	7.98	0.005
Non-family member	36 (56.3)	16 (30.2)		

### Trauma Symptom Differences

Cumulative trauma symptoms scores were higher for children who were poly-victimized as compared to children exposed to CSA only, *t*_(115)_ = −2.24, *p* = 0.03 (See Table 5). Examining specific trauma symptoms, physical aggression, sexual acting out, and substance use were more frequently endorsed for children who were poly-victimized as compared to children exposed to CSA only.

**Table 5:** Differences in trauma symptoms among children exposed to CSA only vs. those who were poly-victimized.

**Characteristic**	**Exposure group**			
	**CSA only (*n =* 64)**	**Poly-victimization (*n =* 53)**		
			***t-value***	***P–value***
Cumulative trauma symptoms, M (SD)	4.72 (2.33)	5.72 (2.47)	−2.24	0.03
Physical aggression, *N* (%)			***χ2***	***P-value***
No	40 (62.5)	22 (41.5)	5.3	0.02
Yes	24 (37.5)	31 (58.5)		
**Oppositional behavior**, ***N*** **(%)**				
No	39 (60.9)	26 (49.1)	1.66	0.20
Yes	25 (39.1)	27 (50.9)		
**Acting out in a sexual manner**, ***N*** **(%)**				
No	52 (81.3)	28 (52.8)	10.83	0.001
Yes	12 (18.8)	25 (47.2)		
**Anxiety or fearfulness**, ***N*** **(%)**				
No	15 (23.4)	9 (17.0)	0.74	0.39
Yes	49 (76.6)	44 (83.0)		
**Nightmares or sleep disturbance**, ***N*** **(%)**				
No	16 (25.0)	21 (39.6)	2.87	0.09
Yes	48 (75.0)	32 (60.4)		
**Changes in weight or appetite**, ***N*** **(%)**				
No	42 (65.6)	31 (58.5)	0.63	0.43
Yes	22 (34.4)	22 (41.5)		
**Preoccupation with abuse**, ***N*** **(%)**				
No	37 (57.8)	24 (45.3)	1.82	0.18
Yes	27 (42.2)	29 (54.7)		
**Feelings of guilt or shame**, ***N*** **(%)**				
No	29 (45.3)	22 (41.5)	0.17	0.68
Yes	35 (54.7)	31 (58.5)		
**Sad or withdrawn behavior**, ***N*** **(%)**				
No	26 (40.6)	24 (45.3)	0.26	0.61
Yes	38 (59.4)	29 (54.7)		
**Thoughts/attempts of self-harm or suicide**, ***N*** **(%)**				
No	45 (70.3)	30 (56.6)	2.37	0.12
Yes	19 (29.7)	23 (43.4)		
**Substance use**, ***N*** **(%)**				
No	61 (95.3)	43 (81.1)	5.90	0.02
Yes	3 (4.7)	10 (18.9)		

### Protective Factor Differences

Differences in protective factors for children who were poly-victimized vs. CSA only are presented in Table 6. Children who were poly-victimized children had lower mean levels of protective factors than children exposed to CSA only, *t*_(85)_ = 4.67, *p* < 0.001. Overall, children who were poly-victimized were less likely to have personal skills, peer-support, social skills, caregiver physical support, caregiver emotional support, and educational support, compared to children exposed to CSA only.

**Table 6:** Differences in protective factors among children exposed to CSA only vs. those who were poly-victimized.

**Characteristic**	**Exposure group**			
	**CSA only (*n =* 64)**	**Poly-victimized (*n =* 53)**	***t-value***	***P-value***
Cumulative protective factors, M (SD)	5.53 (0.86)	4.30 (1.47)	4.67	<0.001
			***χ2***	***P-value***
**Personal skills**				
No	3 (5.2)	16 (32.0)	13.33	<0.001
Yes	55 (94.8)	34 (68.0)		
**Peer support**				
No	4 (9.1)	9 (25.7)	3.92	0.048
Yes	40 (90.9)	26 (74.3)		
**Social skills**				
No	5 (9.8)	15 (32.6)	7.69	0.006
Yes	46 (90.2)	31 (67.4)		
**Physical caregiving**				
No	2 (3.4)	8 (16.7)	5.51	0.02
Yes	57 (96.6)	40 (83.3)		
**Psychological caregiving**				
No	1 (1.6)	6 (11.8)	4.96	0.03
Yes	61 (98.4)	45 (88.2)		
**Educational support**				
No	3 (6.3)	15 (37.5)	13.10	<0.001
Yes	45 (93.8)	25 (62.5)		

### Examining Factors Associated With Cumulative Trauma Symptoms

Using regression analyses, we examined whether demographic and abuse characteristics, including poly-victimization, were associated with cumulative child trauma symptoms (See Table 7). In unadjusted analyses, child age, parental abuse history, parental mental health or addictions history, and poly-victimization status were all positively associated with cumulative child trauma symptoms. Protective factors were negatively associated with child trauma symptoms. In adjusted analyses where all significant variables were entered simultaneously, only child age, and cumulative protective factors remained significant. After accounting for all other factors, poly-victimization status was not significantly associated with child trauma symptoms. A total of 18.8% of the variance in cumulative trauma symptoms was accounted for by the adjusted model.

**Table 7:** Unadjusted and adjusted regression analyses for cumulative trauma symptoms.

**Variable**	**B**	**SE**	***P*-value**	**Standardized Beta**	**95% CI**
**Unadjusted analyses**					
Child age	0.16	0.06	0.009	0.24	0.04, 0.28
Child sex	0.28	0.55	0.61	0.05	−0.80, 1.37
Protective factors	−0.65	0.18	0.001	−0.36	−1.00, −0.29
Financial concerns	0.68	0.65	0.31	0.10	−0.62, 1.96
Parental abuse history	1.18	0.44	0.009	0.24	0.30, 2.05
Parental mental health or addictions history	1.17	0.46	0.01	0.23	0.25, 2.09
Abuse invasiveness	0.25	0.24	0.29	0.10	−0.22, 0.73
Relationship to perpetrator	−0.52	0.45	0.25	−0.11	−1.42, 0.37
Poly–victimization status	0.99	0.45	0.03	0.21	0.12, 1.88
**Adjusted analyses**					
Child age	0.13	0.07	0.06	0.20	−0.006, 0.270
Protective factors	−0.69	0.21	0.001	−0.39	−1.10, −0.28
Parent abuse history	0.44	0.59	0.45	0.09	−0.73, 1.61
Parental mental health or addictions history	0.16	0.59	0.78	0.03	−1.00, 1.33
Poly–victimization status	−0.27	0.56	0.63	−0.06	−1.39, 0.85

*For the unadjusted analyses, each variable was entered separately into the multiple regression model. For the adjusted analyses, significant variables from the unadjusted analyses were simultaneously entered into the multiple regression model. A total of 18.8% of the variance in cumulative trauma symptoms was accounted for by the adjusted model*.

## Discussion

Extant literature has documented the detrimental effects of sexual abuse on children's mental health and emotional well-being (2–4). However, there is significant variability in the individual characteristics and presenting concerns of children referred to mental health services following exposure to CSA and clinicians can benefit from indicators that can help identify which children are at increased risk for poor outcomes or require additional supports. Disentangling whether CSA in addition to other forms of maltreatment conveys additional risk on children's presenting trauma symptoms and protective factors at the outset of child trauma treatment could help inform treatment planning and resource allocation for families. Using a clinical sample of children exposed to CSA, the current study builds on prior research by examining risk factors for poly-victimization, differences in protective factors for poly-victimized children as compared to children exposed to CSA only, and exploring the relative contribution of poly-victimization to child trauma symptoms. We found that lower levels of protective factors and parental history of child abuse were associated with poly-victimization. When examining differences in demographic and abuse characteristics for children exposed to poly-victimization vs. CSA only, we found overall higher levels of family risk, including parental abuse history, parental substance use, and parental mental health difficulties. Children exposed to poly-victimization had a higher number of trauma symptoms and a lower number of protective factors. Although exposure to poly-victimization was associated with greater trauma symptoms, these findings were no longer significant after accounting for protective factors and child age. We discuss our findings and their clinical implications below.

We found that lower levels of protective factors and parental history of child abuse were associated with poly-victimization status. Generally, it may be that children who are exposed to poly-victimization experience more household family risk and fewer supports than children exposed to CSA only. According to the cycle of maltreatment hypothesis, parents who were exposed to child maltreatment themselves are more likely to have children who also experience maltreatment (37–39). Indeed, a previous meta-analysis demonstrated support for this hypothesis, including the specific transmission of neglect, physical, emotional, and sexual abuse across generations (37). Parents who have been abused themselves may have had sub-optimal models for parenting skills and strategies. In families with histories of maltreatment, there may also be increased likelihood of exposure to a family member who may perpetrate sexual abuse and a lack of adequate supervision on the part of the parent, who may have an altered sense of safety based on their own abuse experiences. Parent experiences of mental illness and substance use may also confer risk for child exposure to abuse through compromised parenting quality, reduced supervision, and exposure to environments where maltreatment is more likely to occur. For example, parent psychopathology, such as depression, may disrupt normative parenting practices, which in turn, can increase likelihood for child maltreatment (40). Of note, parents with their own abuse histories may be at increased risk for mental health difficulties, further perpetuating the cycle of maltreatment (41). Parental substance use may also compromise parenting quality, such that previous research has found an inverse relationship between parental drug use and parental involvement (42). Overall, this suggests that, through compromised parenting involvement and quality, parent experience of mental illness and substance use can increase the likelihood that the child is exposed to unsafe situations (43), which may increase risk for exposure to multiple forms of child maltreatment.

Interestingly, child age, sex, and financial concerns were not significantly associated with poly-victimization after accounting for other variables. These findings differ from previous research that found that poly-victimized children in Korea were more likely to be older and be from families with lower household income than children exposed to CSA only (22). One reason for these findings may be that our measurement of financial concerns did not fully capture the financial needs and experiences of families. We may have found differences had we asked families to report on their household family income on a gradient. Given that financial resources have been shown to play a role in symptom reduction for individuals receiving psychological treatment following CSA (44), future research should include more fine-grained measures of family income.

In addition to differences in parental characteristics, we found that at treatment entry, cumulative trauma symptom scores were higher among poly-victimized children compared to children exposed to CSA alone. This finding is consistent with our hypotheses and is in line with the notion of cumulative risk, which suggests that exposure to a greater number of risk factors leads to greater negative outcomes (18). Whereas, previous research has found an association between the cumulative maltreatment and child trauma symptoms (29), the current study demonstrates this association in a clinical sample of youth directly receiving treatment for maltreatment experiences. Mechanistically, exposure to multiple forms of stress can lead to physiological dysregulation, or elevated allostatic load on the body, which in turn, can increase risk for mental health difficulties, such as trauma symptoms (45, 46). Exploring various trauma symptoms, the present research found that poly-victimized children exhibited greater externalizing difficulties, including physical aggression, sexual acting out, and substance use, but not internalizing symptoms (i.e., sadness, anxiety, shame/guilt) compared to children exposed to CSA only. Exposure to additional forms of abuse, such as physical abuse and domestic violence, may increase the propensity for externalizing difficulties. For example, previous research has demonstrated that children exposed to both domestic violence and physical abuse exhibited more externalizing symptoms compared to children exposed to either domestic violence or physical abuse alone (47). Additionally, these findings may support the notion that cumulative risk leads to cumulative outcomes (48). That is, increased exposure to maltreatment types may broaden the potential symptom outcomes that children experience, which has important implications for the delivery of trauma-based interventions.

An important and novel finding from the current study was that children exposed to poly-victimization were less likely to have any protective factors present across the social ecology at the outset of treatment. That is, children who were poly-victimized were less likely to have individual, familial, or school-level factors present. This is concerning as protective factors such as individual coping skills, peer relations, and support from a caregiver have been found to be associated with adaptive outcomes following exposure to maltreatment (49) and are associated with lower levels of trauma symptoms as well as a decreased likelihood of dropping out of treatment prematurely (20, 29). Indeed, we found that protective factors were associated with cumulative trauma symptoms at the outset of treatment in this sample, above and beyond poly-victimization status. Thus, identifying protective factors and supporting families to bolster or increase the protective factors that are present for the child are important treatment targets in trauma therapy, regardless of poly-victimization status.

### Limitations

The current study should be considered in the context of some limitations. First, in order to provide clinically and ecologically valid findings, we employed retrospective file review methodology for the current study. However, this means that we were limited by the information that was present and recorded in the patient file. For example, there was limited information provided with regards to the child's cultural or religious supports. Future research that includes self-report questionnaires that specifically ask about diverse protective factors across the social ecology will be informative. Second, the presence of trauma symptoms was reported by the individual referring the child, meaning that this information was not provided via child or adolescent self-report. Previous research has demonstrated that a child's self-report of trauma symptoms may be more strongly associated with a diagnosis of post-traumatic stress disorder than a parent's report (50). Thus, a goal for future studies would be to obtain both parent and child reports of trauma symptoms. Third, child abuse exposure was also determined via self-report from referral sources and not explicitly obtained from child self-report or disclosures. Future research would benefit from detailed interviews to ascertain abuse exposures. Lastly, although we obtained information on parent exposure to childhood maltreatment, the type of maltreatment was not specified. Future research investigating the role of different forms of parent exposure to child maltreatment and risk for intergenerational transmission may be useful for prevention.

## Conclusion and Clinical Implications

Children who are exposed to CSA are at considerable risk for experiencing disrupted physical and mental health trajectories (2, 6). Of these children, those who have experienced poly-victimization present with particularly challenging familial situations, increased symptom severity, and fewer protective factors. Taken together, our study suggests that both the individual and family-level risk for children who have been poly-victimized are elevated and warrant attention as part of trauma treatment. From a clinical perspective, acknowledging the potentially heightened level of individual and familial-level risk that may exist for children who have been poly-victimized is critical. For example, undertaking a thorough assessment that assesses a wide array of potential victimization experiences and their impact can be helpful (51). A novel contribution of the current study is that a lack of protective factors in the child's developmental ecology confers greater risk for poly-victimization and elevated child trauma symptoms. Thus, assessing and identifying protective factors that exist both inside and outside the home may provide critical information with regards to treatment planning. Questionnaires that specifically target protective factors across the child's social ecology, including at school and in the community (34), may help a clinician to identify where collaborative efforts across systems may be helpful. Additionally, in families where parents and caregivers have had their own previous trauma or are actively managing mental health or substance use issues, it is critical to ensure that these needs are met in order for the parent to be available and supportive to their child as part of trauma treatment (52). Future research that is longitudinal, multi-informant, and considers a broad array of protective factors and their potential mechanisms is needed.

## Data Availability Statement

The datasets presented in this article are not readily available because of confidentiality of participant data. Requests to access the datasets should be directed to sheri.madigan@ucalgary.ca.

## Ethics Statement

The studies involving human participants were reviewed and approved by University of Calgary Research Ethics Board. A waiver of consent was obtained due to the archival file review nature of the data in accordance with institutional requirements.

## Author Contributions

NR and SM: conceptualization, methodology, formal analysis, and supervision. NR, SM, and CH: data curation, project administration, and funding acquisition. NR, SM, and JZ: writing—original draft preparation. NR, SM, JZ, and CH: writing—review and editing. All authors contributed to the article and approved the submitted version.

## Funding

NR received post-doctoral funding from Alberta Innovates. SM receives support from the Canada Research Chairs program. JZ receives graduate student funding from the Social Sciences and Humanities Research Council. This project was supported by a Social Sciences and Humanities Research Council Partnership Engage Grant.

## Conflict of Interest

The authors declare that the research was conducted in the absence of any commercial or financial relationships that could be construed as a potential conflict of interest.

## Publisher's Note

All claims expressed in this article are solely those of the authors and do not necessarily represent those of their affiliated organizations, or those of the publisher, the editors and the reviewers. Any product that may be evaluated in this article, or claim that may be made by its manufacturer, is not guaranteed or endorsed by the publisher.
